# The evolution and heterogeneity of neutrophils in cancers: origins, subsets, functions, orchestrations and clinical applications

**DOI:** 10.1186/s12943-023-01843-6

**Published:** 2023-09-07

**Authors:** Siyao Liu, Wenchuan Wu, Yueshan Du, Hanlin Yin, Qiangda Chen, Weisheng Yu, Wenquan Wang, Jun Yu, Liang Liu, Wenhui Lou, Ning Pu

**Affiliations:** 1 Department of Pancreatic Surgery, Zhongshan Hospital, Fudan University, Shanghai, 200032 P.R. China; 2 Cancer Center, Zhongshan Hospital, Fudan University, Shanghai, 200032 China; 3 Department of General Surgery, Zhongshan Hospital, Fudan University, Shanghai, 200032 China; 4 Departments of Medicine and Oncology, Johns Hopkins University School of Medicine, Baltimore, MD 21287 USA

**Keywords:** Neutrophils, TANs (tumor-associated neutrophils), Heterogeneity, Origins, Subsets, Functions, Orchestrations, Clinical applications

## Abstract

Neutrophils, the most prevalent innate immune cells in humans, have garnered significant attention in recent years due to their involvement in cancer progression. This comprehensive review aimed to elucidate the important roles and underlying mechanisms of neutrophils in cancer from the perspective of their whole life cycle, tracking them from development in the bone marrow to circulation and finally to the tumor microenvironment (TME). Based on an understanding of their heterogeneity, we described the relationship between abnormal neutrophils and clinical manifestations in cancer. Specifically, we explored the function, origin, and polarization of neutrophils within the TME. Furthermore, we also undertook an extensive analysis of the intricate relationship between neutrophils and clinical management, including neutrophil-based clinical treatment strategies. In conclusion, we firmly assert that directing future research endeavors towards comprehending the remarkable heterogeneity exhibited by neutrophils is of paramount importance.

## Introduction

Neutrophils, the predominant common type of polymorphonuclear leukocytes [1], hold a prominent position in the realm of inflammation, particularly acute inflammation, serving as a fundamental part of the innate immune system [2]. In recent years, there has been a surge of interest among researchers in investigating the multifaceted functions and remarkable heterogeneity of neutrophils within the context of cancer. Neutrophils have two specific characteristics: a short lifespan and an inability to proliferate, which distinguishes them from other immune cells. These characteristics once led people to underestimate their role in cancers, which are chronic malignant diseases [3]. However, as our understanding of neutrophils has grown more profound, investigations have substantiated that their lifespan can extend up to 5.4 days. Neutrophils not only constitute the preeminent population of circulating cells within the human bloodstream but also exhibit a remarkable propensity for infiltration into the intricate milieu of the tumor microenvironment (TME) in substantial quantities [4, 5]. Thus, researchers have recognized the pivotal role of tumor-associated neutrophils (TANs) within the TME, and substantial endeavors have been devoted to elucidating their potential as therapeutic targets [6].

Understanding the nature of immune cells in cancer is a gradual process, with earlier investigations primarily centered around adaptive immune cells. There is a generally accepted classification of T helper cell as the “Th1/Th2” paradigm, which subsequently directs the grouping of other immune cell populations [7]. Nevertheless, it has been proven that solely targeting the activation of T cells to enhance the immune system’s cytotoxic potential is insufficient to address the underlying challenge of immunosuppression in the context of cancer [8]. As the understanding of the TME deepens, the research emphasis has shifted from adaptive immunity to innate immunity. Amongst the key players, macrophages, which originate from myeloblasts similar to neutrophils, have garnered significant attention. These macrophages can be divided into distinct polarization states known as “M1/M2”, partially resembling the Th1/Th2 paradigm. M1/M2 refer to the polarization state of macrophages as having anti-tumor and pro-tumor potential, respectively [9, 10]. Based on this background, and considering the similarities between neutrophils and macrophages as myeloid cells, both of which play vital roles in maintaining immunological homeostasis in inflammation and cancers, there has also been a proposition to categorize TANs into N1/N2 groups [11, 12]. As researches on TANs have advanced, this hypothesis has been further substantiated, as neutrophils exhibit a dual function that is intricately linked to the prognosis [11, 13, 14].

However, recent research suggests that the simplistic dichotomy of immune cells in cancer may not provide a comprehensive depiction of the entire landscape. Studies have unraveled the heterogeneity within T cells, revealing the existence of different subsets of less-differentiated T cells that possess extended lifespans and greater potential to overcome T cell inhibition [15, 16]. Furthermore, various subsets of neutrophils have been identified in cancer, exhibiting discrepancies in surface markers and functions [17]. Given the heterogeneity and remarkable plasticity of TANs within the TME, conducting an accurate subset analysis of TANs has emerged as a significant research focal point [18]. Nevertheless, it is important to note that neutrophils in cancer go beyond TANs and encompass numerous subsets in both bone marrow and circulation. TANs can be perceived as a consequence of neutrophil development and subsequent infiltration into the TME. To date, the extensive heterogeneity of neutrophils in cancer has remained a topic warranting further investigation.

Numerous studies have demonstrated that neutrophils in cancer exhibit the biological heterogeneity in their lifespan, prompting the need for a fresh investigation approach. Does heterogeneity present in neutrophil progenitors result in specific neutrophil progeny within circulation and contribute to tissue heterogeneity? Considering the different functions of neutrophil subsets in the TME, is targeting all TAN subsets a viable therapeutic strategy? While research has predominantly focused on targeting immunosuppressive or other pro-tumor TAN subsets, is it feasible to control and harness their anti-tumor functions? It is worth noting that the answers to these questions may vary depending on tumor types and stages of disease progression, further complicating the matter [19]. Therefore, future studies aimed at addressing these questions will not only help elucidate the new roles of neutrophils in cancer progression but also facilitate the development of new treatments from a broader perspective of neutrophil lifespan, rather than exclusively within the confines of the TME.

This comprehensive review delved into the entire developmental process of neutrophils in the context of cancer, aiming to interpret the complex phenomenon that correlates neutrophils with the occurrence and development of cancer by taking into account their heterogeneity. We provided an in-depth exploration of the past and present lives of TANs, including their sources, factors influencing chemotaxis and activation, as well as their phenotypes within the TME. Furthermore, we systematically outline the intimate relationship between neutrophils and prognosis, along with neutrophil-based therapeutic strategies. Overall, we firmly believe that directing research efforts towards unraveling the remarkable heterogeneity of neutrophils holds paramount significance, as it bears crucial implications for the development of neutrophil-related therapeutics.

## Heterogeneity among the “previous lives” of TANs

### The development of neutrophils in bone marrow

Bone marrow is the origin of all mature neutrophils. Neutrophil precursors continuously differentiate through different hematopoietic progenitor stem cells until they mature. Density gradients, followed by Giemsa staining, are widely accepted methods to isolate and identify granulocyte precursors. Morphological observation can be the second step to identify granulocytes based on their special characteristics, including cell size, nuclear condensation, and granule content [20]. Multipotent granulocyte-monocyte progenitors (GMPs), which originate from myeloid progenitors, are the precursor cells of neutrophil precursors [21]. Thus, GMPs could be considered as the upstream progenitor of all neutrophils in hematopoiesis. Following the neutrophil developmental process, promyelocytes are the adjacent downstream cells and start to express the neutrophil-specific lineage marker CD66b [1]. The subsequent process can be thought of as “neutrophil maturation” instead of “neutrophil formation”, which includes several cell stages: myelocytes, metamyelocytes, band cells, and segmented neutrophils in sequence. During this process, the expression of CD11b and CD16 is upregulated, along with CD66b. The process of neutrophil differentiation and maturation in the bone marrow has been extensively studied in the past decades. The surface markers for each differentiation stage have been clearly identified, which may provide inspiration for research in other physiological and pathological environments.

Recently, state-of-the-art technologies such as single-cell RNA sequencing and mass cytometry have garnered substantial attention in the field of scientific research, providing a comprehensive description of genomic states. Consequently, our understanding of the developmental and maturational process of neutrophils has witnessed significant advancements [22, 23], giving rise to the concept of heterogeneity of neutrophils in the bone marrow under pathological conditions [24]. However, in the context of cancer, the full picture remains elusive in cancers. Commencing from the most upstream of neutrophil differentiation, the unipotent early-stage neutrophil progenitor (eNeP) has been discerned as a subset of GMPs in both humans and mice; Notably, eNeP exhibits unique characteristics that set it apart from other unipotent progenitors responsible for the development of granulocytes and monocytes [25]. Subsequently, a novel human neutrophil progenitor subset was discovered, representing the beginning stream cell of GMPs by detecting the co-expression of CD71 and CD117 subsets. Notably, CD71 has been identified as a proliferative marker of immature neutrophils [26]. In another study, a committed proliferative neutrophil precursor (pre-neutrophil, preNeu) was identified as a subset of promyelocytes and was considered to be located downstream of eNePs in the neutrophil differentiation pathway. Researchers believed that preNeu played a key role in neutrophil development due to its unique potential to differentiate into both non-proliferating immature neutrophils and fully mature neutrophils [27]. Nevertheless, the precise influence of transcription levels on the heterogeneity of neutrophil progenitors and their subsequent progeny remains uncertain. Xie et al. [24] highlighted that the effects of transcription factors may be definitive to neutrophil progenitors, particularly in the context of pathological conditions, which could even bring heterogeneity to their progeny, as evidenced by mouse models. Furthermore, a recent study revealed the presence of a dominant single developmental spectrum in transcriptomic patterns encompassing the diverse range of neutrophil heterogeneity observed during inflammation conditions [28].

Recent studies using single-cell RNA sequencing and mass cytometry have identified several subsets of proliferative neutrophil progenitors that can enter circulation in both humans and mice under cancerous conditions. However, despite these advancements, our current understanding of the intricate processes involved in the entry of abnormal and proliferative neutrophils into the circulation from the bone marrow remains incomplete. Moreover, the mechanisms governing their subsequent recruitment into the TME and their consequential role in in cancer immunity remain inadequately elucidated. The heterogeneity exhibited by neutrophils arising from different subsets of neutrophil progenitor cells, along with their commonalities in the TME, the dynamic interplay between progenitor cells and progeny, and the regulatory factors governing these processes collectively serve as pivotal focal points linking neutrophil developmental biology and tumor immunology.

**Fig. 1:**
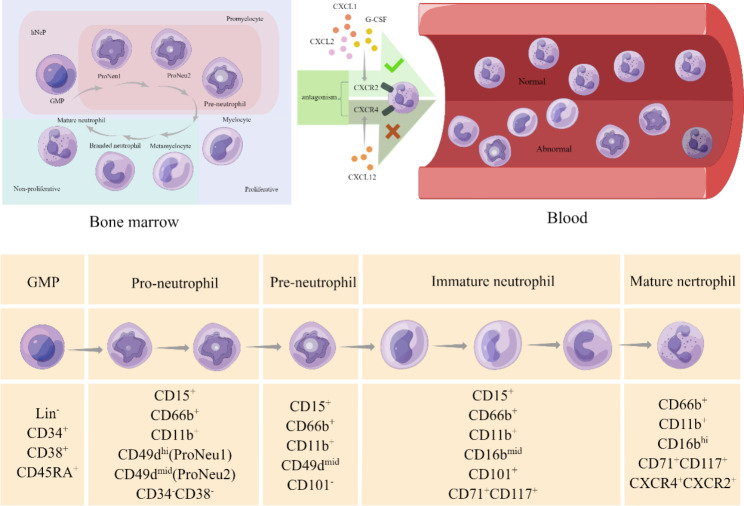
**The evolution of neutrophils in humans.** In humans, neutrophils originate from GMPs residing within the bone marrow, which are characterized by the expression of CD34, CD38, and CD45RA [29, 30]. Subsequently, these GMPs differentiate into pro-neutrophils (pro-neutrophil 1 and pro-neutrophil 2) and pre-neutrophils, expressing biomarkers such as CD11b, CD66b, and CD15 [31, 32]. This specific stage of neutrophil development, characterized by the presence of immature neutrophils exhibiting relatively high levels of biomarkers such as CD11b, CD16b, CD71, and CD117, has been duly acknowledged [26, 31, 33]. Ultimately, neutrophils that express chemokine receptors (CXCR)4 and CXCR2 undergo final maturation and are subsequently released into circulation. The C-X-C Motif Chemokine Ligand (CXCL)12 expressed by bone marrow stromal cells activates the neutrophil receptor CXCR4 to retain it within the bone marrow. While under the stimulation of G-CSF, endothelial cells in the bone marrow upregulate CXCL2 expression to activate CXCR2 signaling, thereby facilitating the releasing of neutrophils from the bone marrow into circulation [34, 35, 36]. Conversely, under pathological circumstances, the presence of mature neutrophils exhibiting abnormal biomarkers or immature neutrophils within the peripheral blood of humans has been reported [37, 35, 38]

### Neutrophil subsets in circulation

Under normal physiological conditions, mature neutrophils are released from the bone marrow and enter the bloodstream due to the stimulation of granulocyte colony-stimulating factor (G-CSF) [36]. Notably, CXCR4 and CXCR2 are the two most critical markers governing this process. Specifically, only fully differentiated neutrophils that express CXCR2 are permitted to enter the human bloodstream and subsequently infiltrate corresponding tissues [34, 37]. Under normal conditions, neutrophils gradually lose CD62L (also known as L- selectin) and express CD11b and CXCR4 within a timeframe of approximately 6 h [39]. Additionally, it is crucial to take into account the phenomenon of neutrophil aging when discussing surface markers, not to mention the rhythmic modulation of neutrophil clearance, thereby introducing an additional layer of complexity to the overall process [40]. As mentioned above, the physiological heterogeneity of neutrophils in circulation already poses challenges in their study. In the context of cancer conditions, this complexity is further amplified. Recently, Zhu et al. [41] unveiled the existence of several neutrophil subsets within the circulation of cancer patients, characterized by divergent functional properties.

The initial investigation into circulating neutrophils in cancer patients employed density centrifugation to separate neutrophils into three distinct subsets [42]. Subsequent studies highlighted that the abundant presence of low-density neutrophils in circulation could potentially stem from the transforming growth factor beta (TGF-β)-dependent differentiation of high-density neutrophils. Notably, these low-density neutrophils (LDNs) are capable of suppressing CD8^+^ T cell proliferation, thereby promoting tumor growth [42, 43]. Given the association of elevated levels of LDNs with a poor prognosis in cancer patients, it is imperative to further investigate LDN levels along with other biomarkers such as neutrophil-platelet aggregates (NPAs) as potential novel prognostic indicators [44]. On the other hand, these low-density neutrophils exhibit a close association with immature neutrophils. Studies have revealed a distinct population of immature neutrophils in the spleen, possessing both proliferative capacity and immunosuppressive properties [43, 45]. Notably, these immature neutrophils, including the earliest neutrophil progenitors listed above [25, 26], are released into circulation and may facilitate the malignant behavior of tumors. CD71-expressing eNePs have been identified in the blood of melanoma patients [25] as well as lung cancer patients [26]. Additionally, preNeus, detected both in peripheral blood and tumors of tumor-bearing mice, may have the potential to significantly promote tumor growth [43]. These findings substantiate the concept of “emergency granulopoiesis,“ highlighting enhanced proliferation of myeloid precursor cells in the bone marrow under pathological conditions. The excessive presence of immature neutrophils in circulation could perceived as a response to the tumor [46].

### Recruitment of neutrophils from circulation to the TME

One classical approach in investigating the recruitment of neutrophils by tumors is to examine this phenomenon from an inflammatory perspective. Previous studies have confirmed the intricate association between inflammation and cancer [47]. Notably, well-established models, exemplified by the inflammation-melanoma model in zebrafish, have been widely employed to explore the recruitment of neutrophils during cancer initiation [48]. Researchers have demonstrated that both inflammation and tumors share some common mechanisms to recruit neutrophils. In this process, damage-associated molecular patterns (DAMPs) and certain chemokines, such as Interleukin (IL)-8, play pivotal roles, while the binding of IL-8 to its receptors. CXCR1/2 contributes to the initial recruitment of neutrophils [49]. On the other hand, a study has revealed how neutrophils migrate from sterile inflammatory sites to the lung and bone marrow [50]. Studies have confirmed a clear association between the infiltration of neutrophils in the lungs and the metastasis of lung tumors. Considering that the lungs as one of the primary destinations for neutrophil migration during sterile inflammation, it is reasonable to speculate that specialized neutrophil subsets may play a dual role in both inflammation and cancer migration, or that there might be shared mechanisms at work. Therefore, interpreting neutrophils from the perspective of inflammation is undoubtedly helpful for understanding tumor metastasis [51, 52]. Hence, the interpretation of neutrophils through the lens of inflammation is likely to provide great inspiration for unraveling the complexities of cancer metastasis.

As mentioned above, colony-stimulating factors (CSFs) play a pivotal role in stimulating the proliferation and differentiation of neutrophils in the bone marrow. Notably, this mechanism is also exploited by tumors to recruit neutrophils into the TME [53]. Numerous studies have confirmed the phenomenon whereby metastatic tumors secrete large amounts of G-CSF, inducing robust chemotaxis of neutrophils from the circulation. A large proportion of these recruited neutrophils are immature and immunosuppressive, consequently fostering the progression of cancer metastasis [53–55]. Further studies have unveiled the downstream signaling of G-CSF signal, which is mediated by IL-23 and IL-17 secreted by other immune cells present within the TME, such as macrophages and T cells [56, 57]. Considering the potential T cell suppressive effect of G-CSF, along with its close relationship with tumor progression, further studies are warranted to fully understand the therapeutic potential of G-CSF targeting [56]. In the recruitment of TANs, another crucial factor lies in the interaction between the surface marker of neutrophils, CXCR2, and its ligands IL-8(CXCL8). These ligands are known to be abundantly secreted in most cancers and play a pivotal role in regulating the recruitment and functions of TANs [58, 59]. Notably, blocking CXCR2 has demonstrated a significantly reduction in the recruitment of neutrophils to tumors and has shown promise in increasing the efficacy of chemotherapy [60]. Other studies have highlighted the substantial impact of CXCR2 inhibitors in reversing immunosuppression within the TME, leading to enhanced activation of T cells and improved responsiveness to other immunotherapy, ultimately resulting in a more favorable prognosis [61, 62]. A large-scale retrospective study has provided compelling evidence linking serum IL-8 levels to TAN infiltration and the therapeutic response to immunotherapy, thereby confirming the important role of CXCR2-IL-8 as a ligand-receptor pair governing TAN recruitment within the TME [63]. Ongoing research on TAN recruitment is also delving into the intrinsic features of cancer cells, such as their genetic and epigenetic alterations, which have emerged as the primary causes of immunosuppression in the TME [64]. For example, a recent study highlighted that the activation of K-ras and the depletion of Tp53 contributed to the secretion of CXCR2 ligands, leading to the recruitment of immunosuppressive cells and promoting immune tolerance [65]. Similarly, another study analyzed the characteristics of prostate cancer genetics and reported that loss of both Pten and Tp53 resulted in the secretion of CXCL17, which enhances the recruitment of neutrophils and contributes to the immunosuppression of the TME [66].

### Outstanding issues in recruitment of neutrophils to tumors

The exact proportion of these abnormal immature circulating neutrophils that infiltrate the TME and transform into TANs remains unclear. Additionally, the relationship between neutrophil subsets in the bone marrow and those present in the TME has not yet been definitively established. Previous studies have proposed that, in addition to the bone marrow-circulation-TME pathway, the spleen serves as another important source of TANs, where neutrophils may be reprogrammed and induced [67]. Nevertheless, whether TAN differentiation and mobilization occur after circulation or at the bone marrow level remains a subject of debate. A study focusing on lung cancer has identified a special subset of neutrophils that is remotely supplied by osteoblasts highly expressing Siglec-F in the TME in animal models. Notably, this subset has been found to contribute to tumor progression and is reprogrammed within the bone marrow instead of during circulation or within the spleen [68].

Another interesting question is whether the modality by which neutrophils infiltrate the TME and the different upstream subsets are related to the total amount of TANs, a metric closely related to prognosis [69], although the amount varies among different cancers [14]. A prevailing view is that there exists a tissue-specific pattern that may be the determinate factor in neutrophil recruitment, wherein surface markers, chemokines and distinct vascular properties play important role in this process [35, 70]. A recent study have unveiled that the varying amounts and functions of neutrophils among organs, demonstrating an intimate association between the recruitment process and CXCR4 expression on the surface along with CXCL12 enrichment in the lung under physiological conditions, which may provide great inspiration and need to be verified in cancer studies [35].

However, the inherent heterogeneity of neutrophils is evident in studies that aim to localize the upstream of CXCR2 and other pivotal determinants of neutrophil recruitment. Consequently, a fundamental question arises: do these genotypes solely correlate with specific neutrophil subsets or do they possess broader regulatory roles in the recruitment of TANs? Another noteworthy observation on neutrophil recruitment tend to combine T cell activation or response with immunotherapies. These findings lend support to the hypothesis that targeted modulation of pathways implicated in neutrophil recruitment to malignancies may hold promise for enhancing prognosis in cancer patients. Moreover, these studies underscore the significance of comprehensive consideration of remodeling the TME to counteract immune-suppression. Both innate immunity, represented by neutrophils, and adaptive immunity play pivotal roles in this regard.

### Heterogeneity among the “present lives” of TANs

#### Function study from circulating neutrophils to TANs

Studies investigating the functionality of circulating neutrophils have primarily focused on their involvement in the process of metastasis. Notably, one study proposes that neutrophils can facilitate metastatic progression through their interaction with circulating tumor cells (CTCs) in breast cancer [71]. Interestingly, a significant proportion of CTC-associated neutrophils exhibit shared markers as well as expression levels of certain genes with N2 neutrophils, including Arginase1(ARG1), CXCL1, CXCL2, CXCL10, CCL2, CXCR2, and Vascular Endothelial Growth Factor A (VEGFA)[71]. Similarly, a distinct subset of N2 neutrophils expressing AGR2 can be induced by TGF-β1 derived from peripheral blood neutrophils. This particular subset of neutrophils can promote cancer metastasis within the circulation [72]. These identified biomarkers suggest a classification and detection method to precisely determine the heterogeneity and similarities between the circulation and TME. More researches could be carried out in similar directions to verify these phenotypic characteristics of neutrophils across diverse environments.

### The direct interaction between neutrophils and tumor cells

The direct interactions between neutrophils and tumor cells, including both anti-tumor and pro-tumor functions, have long been a focal point in neutrophil research, particularly regarding TANs with their diverse mechanisms. The initial focus was on confirming the anti-tumor function, which was believed to be achieved through various mechanisms, including direct killing via the production of reactive oxygen species (ROS) whose killing function is dependent on tumor cell expression of TRPM2[18, 73] and reactive nitrogen species (RNS) where MET is necessary, as well as antibody-dependent cytotoxicity (a newly discovered mechanism called “trogoptosis“ [74]). Consequently, G-CSF, known for its ability to mobilize and recruit neutrophils, was initially considered an ideal anti-tumor therapeutic agent [75, 76]. However, the pro-tumor function also plays an important role within the TME, including genotoxicity mediated by ROS and RNS [77, 78], cytokine production (e.g., neutrophil elastase (NE) [79], prostaglandin E2 (PGE2) [80], TGF-β&TNF-α [81], among others. ROS and RNS themselves exhibit a dual role in cancers, encompassing both pro- and anti-tumorigenic signaling, modulation of metabolism, facilitation of cancer cell proliferation and survival, as well as induction of cancer cell death, angiogenesis, and DNA damage [82, 83]. Moreover, ROS and RNS can modulate neutrophil migration, adhesion, and even their life span [84]. In vitro studies have successfully induced the dual functions of neutrophils by using interferon-gamma (IFN-γ) and tumor necrosis factor-alpha (TNF-α) to convert N2 neutrophils into N1 neutrophils [85]. Furthermore, type I interferon (type I IFN) has been reported to modulate neutrophil phenotype towards an anti-tumor function in both mouse and human studies. Specifically, a deficiency in IFN-β results in the predominance of tumor-promoting neutrophils within intratumor and lung metastatic lesions [86]. Recent investigations have also sought to modify the immune microenvironment in order to influence the phenotypic alterations of neutrophils. Yunhao Wang et al. acidified the tumor immune microenvironment, resulting in increased captopril release, leading to the polarization of pro-tumor N2 phenotype neutrophils into an anti-tumor N1 phenotype [87]. Although the potential for functional studies is currently limited, these functions have gained recognition as downstream outcomes, serving as endpoints for upstream phenotypic induction studies. The investigation into the mechanisms underlying polarity changes in neutrophils remains an ongoing and perennial topic of research.

**Fig. 2:**
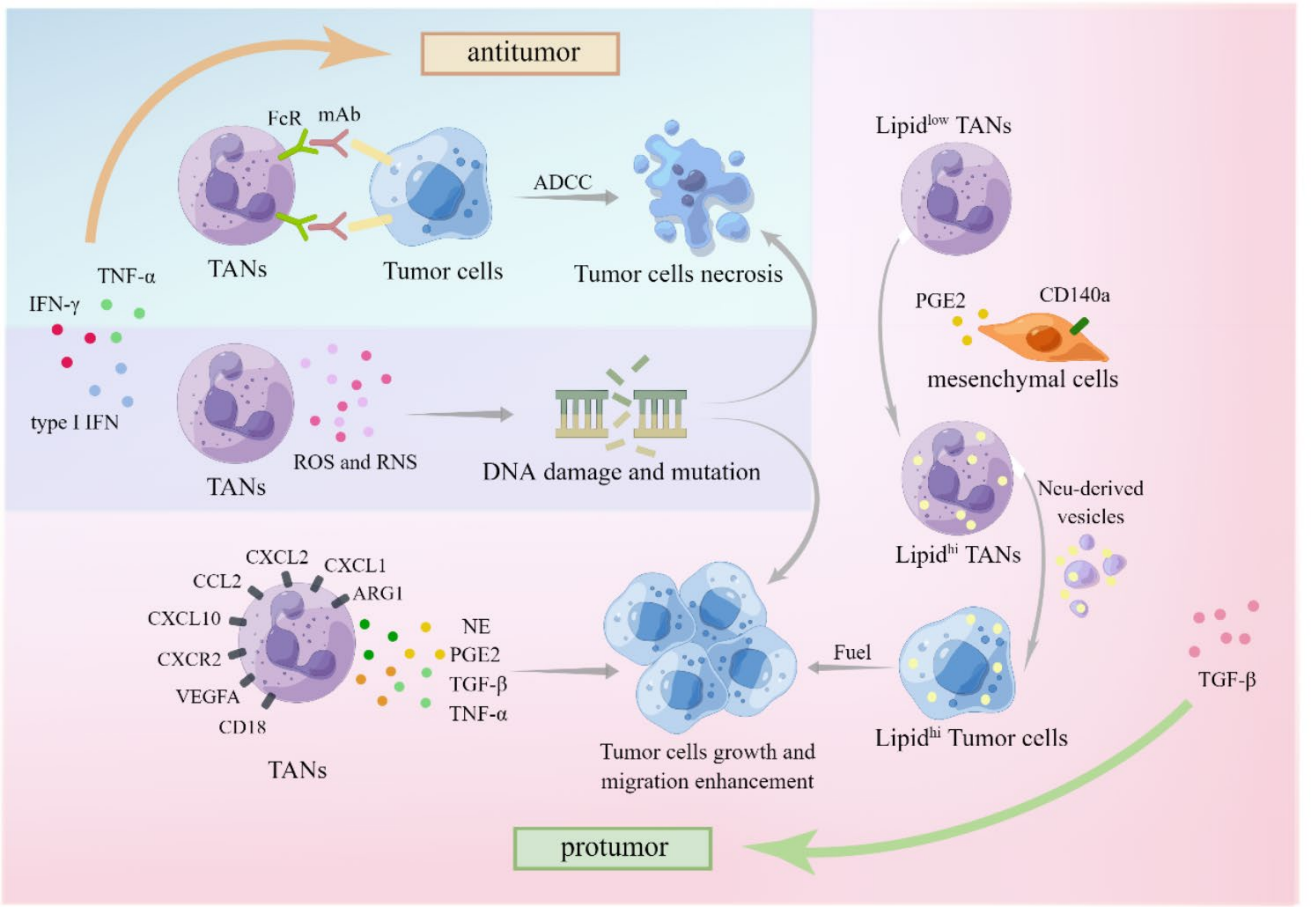
**Direct interaction between TANs and tumor cells.** Interactions between tumor cells and TANs typically lead to two outcomes: promotion or suppression, with the former involving necrosis or growth. The former one can be induced by type I IFN, IFN-γ and TNF-α, while the latter one by TGF-β [41, 85]. Necrosis of tumor cells can be induced not only by Antibody-dependent cell-mediated cytotoxicity (ADCC) of TANs through the combination of Fc receptors (FcR) and monoclonal antibodies (mAbs), but also by DNA damage and mutations triggered by ROS and reactive nitrogen species (RNS) [82, 83, 88]. The latter pathway may exhibit a paradoxical effect of promoting tumor growth and migration [82, 83]. Moreover, TANs secrete various molecules that can stimulate tumor growth, such as neutrophil elastase (NE), prostaglandin E2 (PGE2), TGF-β, and TGF-α [79–81]. Notably, mesenchymal cells expressing CD140a also produce PGE2, which contributes to the accumulation of lipid-rich TANs and subsequently fuels tumor growth through lipid oxidation [89]

### The role of TANs in tumor metastasis

The relationship between neutrophils and tumor metastasis has been extensively studied for decades. Initial investigations thirty years ago involved injecting neutrophils in animal models to examine the contribution of neutrophils to metastasis [90]. As research progressed, the close connection between neutrophils and metastasis has been confirmed. Subsequent studies focused on understanding the role of specific molecules within neutrophils in relation to metastasis. Benedicto et al. [91] demonstrated that β2-integrin (CD18) expressed by neutrophils plays a key role and is positively correlated with the metastasis of colorectal cancer. Wculek et al. showed that leukotriene-generating enzyme arachidonate 5-lipoxygenase (Alox5) suppresses pro-metastatic activity in neutrophils, and inhibiting Alox5 in neutrophils may limit tumor metastasis [92].

Recently, a completely novel perspective has emerged, examining the link between neutrophils and tumor metastasis from the standpoint of energy metabolism. A study on lung metastasis in breast cancer suggests that TANs can act as a source of fuel to promote metastasis [89]. This raises questions about whether all types of neutrophil subsets are capable of providing energy or if only specific differentiated TANs possess this ability. Additionally, it remains unclear whether these neutrophils solely supply disseminated tumor cells or also impact carcinoma in situ [93]. Furthermore, the conditions within the TME that trigger TANs to serve as a nutrient source in metastasis, and whether these conditions vary among different tissues, remain unknown. The metabolic specialization of neutrophils may underlie disease pathology and present opportunities for targeted therapeutic interventions. Targeting specific phenotypes of neutrophils may yield promising therapeutic effect [93]. The same study also revealed that resident mesenchymal cells could stimulate infiltrating neutrophils to accumulate neutral lipids in the lung, while the colonization of disseminated tumor cells initiates the energy supply of TANs [89]. Further investigation into the detailed mechanisms involved would aid in the development of new targeted therapies. For instance, how does TAN sense the appearance of tumor metastasis and act accordingly at the molecular level? What are the characteristics of the specific subsets, and how can we precisely target these TAN subsets? In this perspective, researchers could prevent tumor metastasis more efficiently by cutting off one of its energy supplies, which is essential for tumor progression.

### TANs promote tumor-related inflammation by driving angiogenesis

TANs play a significant role in angiogenesis, a crucial process in the TME. Angiogenesis is mainly induced by cytokines secreted by various cells in the TME [94]. Neutrophils secrete several pro-angiogenic cytokines, including prokineticin 2 (Bv8), VEGFA and matrix metalloproteinase-9 (MMP-9) [95, 96]. Although neutrophil functions in angiogenesis and tissue restoration have been observed in models of sterile and ischemic injuries, as well as in tumors, further studies are needed to explore the selective targeting of different neutrophil subsets [97]. Among the TAN subsets, a specific subset with high expression of neutrophil gelatinase-associated lipocalin (NGAL) has been implicated in angiogenesis and tumor progression [98, 99]. In materials engineering research, N2 neutrophil composite hydrogel scaffolds have shown promising results in regulating inflammation and promoting angiogenesis in vivo, providing evidence for targeting neutrophils to regulate angiogenesis in the TME and demonstrating the positive effect of N2 neutrophils on angiogenesis [100]. So far, N2 neutrophil have been identified as the TANs involved in angiogenesis. However, there is limited evidence to support the involvement of N1 neutrophils, as tumor angiogenesis is crucial for cancer metastasis by providing oxygen, nutrients, and metastatic conduits. Vascular normalization therapy is a new research direction that may also involve neutrophils [101, 102]. Noticeably, the concept of N1 and N2 neutrophils, initially proposed in 2009 to distinguish between anti-tumorigenic and pro-tumorigenic neutrophils [12], is an oversimplification of their dual phenotypes. More detailed subsets related to angiogenesis need to be systematically researched. Furthermore, neutrophil extracellular traps (NETs) released by neutrophils contribute to the pathogenesis of various vascular disorders, leading to tumor progression. Lulwah et al. [103] were the first to report NETs have a significant impact on angiogenesis in inflammatory pathology both in vitro and in vivo. The specific mechanism involves the induction of angiopoietin 1 (ANGPT1) and angiopoietin 2 (ANGPT2), which are potential therapeutic targets for angiogenesis [104]. Nicotinamide phosphoribosyltransferase (NAMPT) also contributes a lot in TANs relevant tumor angiogenesis. Relevant research showed NAMPT is involved in CSF3R downstream signaling and is essential for tumorigenic conversion of TANs. The expression of NAMPT is up-regulated in TANs in several cancer and the inhibition of NAMPT effectively suppresses SIRT1 signaling. Thus, there is a impeding of the transcription of pro-angiogenesis genes [105].

With the improvement of research technology, the analysis of the transcription and chromatin landscapes has become more feasible, which is instrumental in uncovering the key molecules and potential targets in angiogenesis-related therapy. Identifying their biomarkers can facilitate us gradually improve our insight into the phenotypes related to angiogenesis in different subsets [43].

**Fig. 3:**
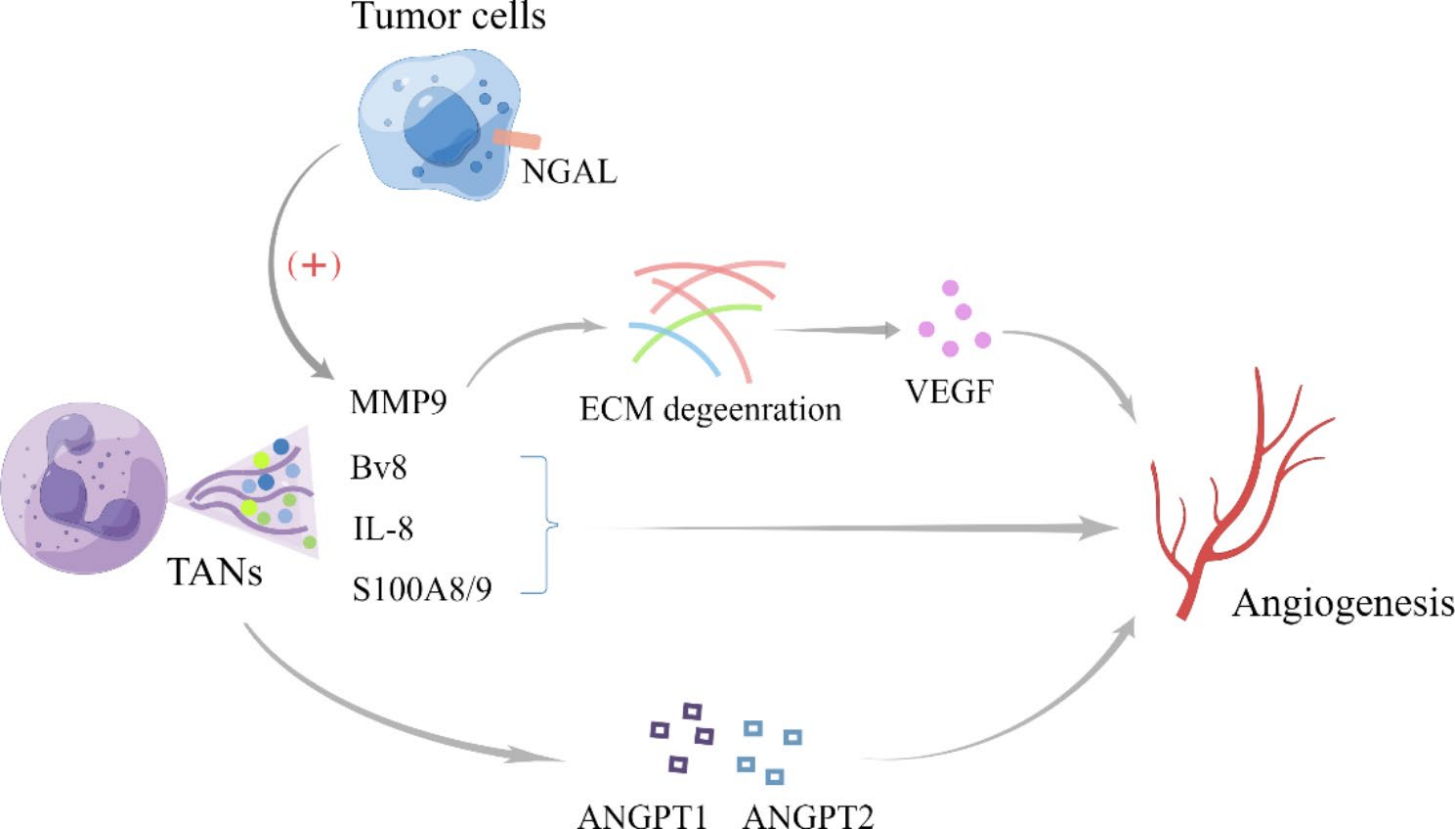
**The multiple tumor-related inflammation pathways of TANs driving angiogenesis.** TANs not only promote angiogenesis through angiopoietin, but also up-regulate the expression of Bv8, IL-8, and S100A8/A9 [104, 106, 107]. The secretion of MMP9 by TANs, in conjunction with a high NGAL expression in tumor cells, can confer a protective effect and promote angiogenesis via extracellular matrix (ECM) degeneration [98, 99]

### The contribution of neutrophils in tumor immunity

Neutrophils also play a dual role in tumor immunity through crucial molecules and interactions with multiple cells, encompassing both innate and adaptive immune cells. These interactions have been extensively studied in the context of inflammation [108]. In the TME, the interaction between TAN and T-cell was initially unveiled. A subset of N1 neutrophil, characterized by the expression of CD86 and HLA-DR, was identified as having antigen-presentation capabilities that enhance the anti-tumor effect of T cells [109]. Another study by Valeria et al. [110] demonstrated that neutrophils enhanced the responsiveness of CD8^+^ T cells to T-cell receptor triggering, thereby improving the OS in colorectal cancer. On the other hand, a previous study pointed out that neutrophils can inhibit the anti-tumor function of T cells and promote tumor metastasis [111]. A recent study also reported an additional regulatory effect on T-cell function: the subset of PD-L1^+^ TANs can suppress T-cell cytotoxicity [112]. Due to the dual regulations to T cells by neutrophils, it is necessary to precisely identify the specific subsets for the development of therapies that enhance T-cell antitumor immunity.

As the depth of research in this field expands, investigators are delving into the intricate interactions between TANs and other components of the innate cells [113]. Most of these interactions are presumed to exert immunosuppressive effects. Zhou et al. [114] highlighted the interaction between TANs and tumor-associated macrophages (TAMs) within the TME, demonstrating their collaborative role in driving the progression of intrahepatic cholangiocarcinoma by activating STAT3. Intriguingly, neutrophils inhibit tumor metastasis without NK cell, while the interplay between NK cells and neutrophils may contribute to the metastasis of breast cancer [115]. Moreover, a recent investigation unveiled that CCL4^+^ TANs recruit macrophages via chemokine secretion, albeit the phenotypic characteristics of these macrophages remain unknown [112]. The dynamic interplay between TANs and macrophages also stimulates macrophage secretion, thereby facilitating T-cell polarization. A special subset of unconventional αβ T cells can produce IFN-γ to kill tumor cells by stimulation of IL-12 as a result of this interaction [116].

A comparative study has scrutinized the phenotypes and immune functions of TANs in circulation and bone marrow, discerning a distinctive subset of TANs in sarcoma characterized by augmented expression of CD11b and CD54, and diminished expression of CD62L. Remarkably, these TANs exhibit an exceptional cytokine profile, encompassing the secretion of cytokines such as Cxcl10, Il23a, and Arg1, which potently bolster the polarization of a subset of CD4^−^CD8^−^ unconventional αβ T cells, showing anti-tumor function [116].

Furthermore, researchers have discovered TANs could also interact with infiltrating B cells in various types of cancer, leading to changes in the behavior of plasma cells accordingly [117]. This interaction is also correlated with the prognosis and survival of cancers [118, 119]. However, the underlying mechanism remains largely unexplored and warrants further research.

Specifically, TANs play a role in mediating B-cell chemotaxis, through the secretion of TNF-α, in conjunction with the presence of CXCL13 or CXCL12 [120]. As the major component of B cells in human, whether follicular B cells directly interact with neutrophils remains unclear. There is evidence suggesting that neutrophils can accumulate in the B-cell zones [121, 122], and secrete B-cell-activating factor (BAFF) through a G-CSF method, which supports the accelerated generation of plasma cells [122]. Besides, TANs have the ability to significantly reduce immunoglobulin production by blocking the BAFF receptor at B cells [123]. Furthermore, there exists a noteworthy population of B cells known as marginal zone (MZ) B cells (also known in humans as IgM memory B cells) in the margin zone of the spleen. These MZ B cells can be activated by the splenic neutrophils via their high B-cell-stimulating/attracting factors secreting, as well as additional NETs [124]. Research has demonstrated that neutrophils are involved in the differentiation of neoplastic B cells as well. This differentiation process can also be mediated by the related molecules mentioned earlier, such as Proliferation-Inducing Ligand (APRIL) [125]. Considering the multiple functions of B cells in anti-tumor immunity, including their direct antitumor effect through antibody-dependent cellular cytotoxicity [126], as well as their ability to activate other immune cells such as T cells and NK cells [127], it is necessary to study whether and how TANs involve in these crosstalk. On the other hand, it poses an interesting question as to whether TANs are relevant to B cells’ immunosuppressive effect, such as lymphotoxin production, tumor angiogenesis, and inhibition of T-cell activity [128–130].

**Fig. 4:**
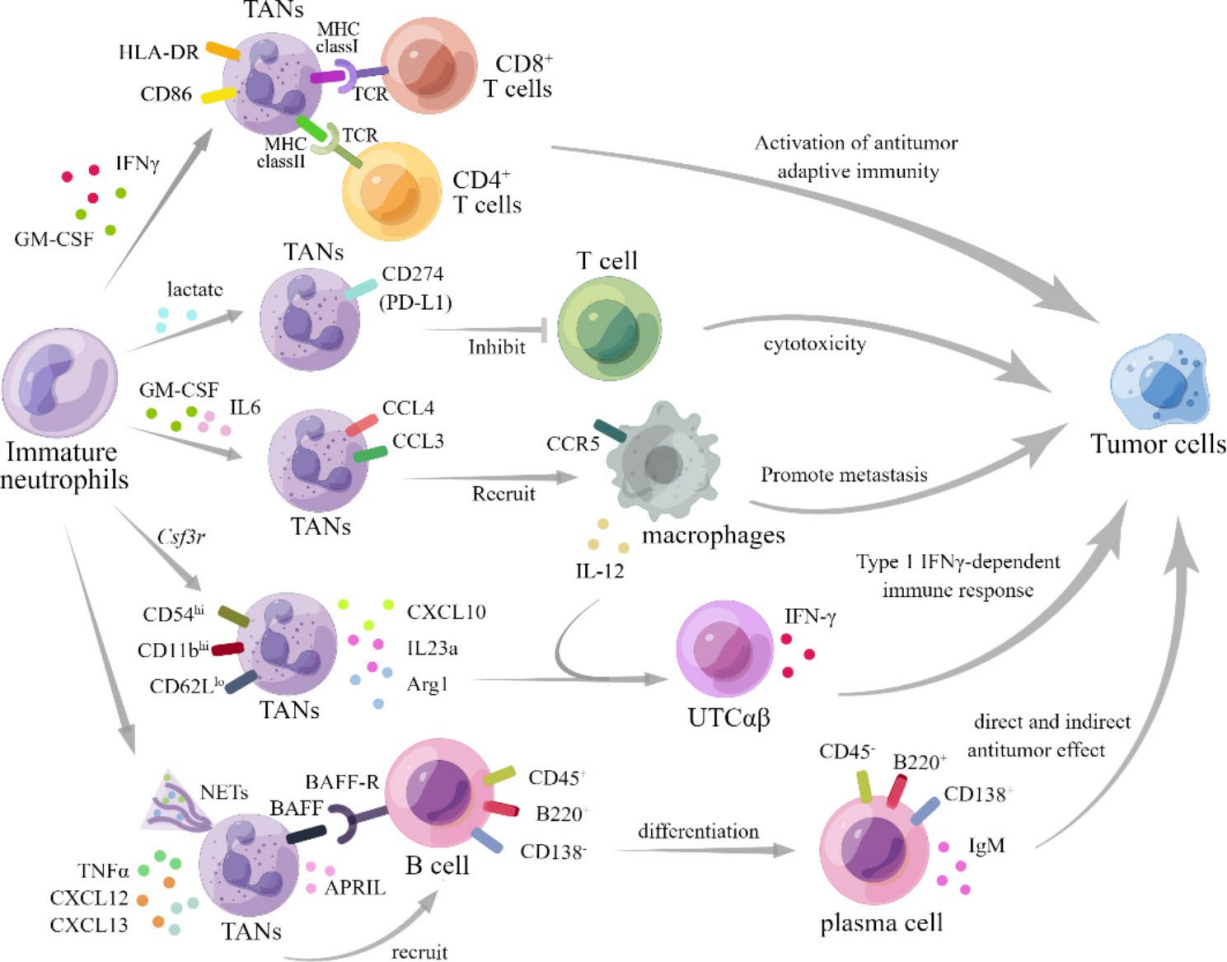
**TANs’ role in tumor immunology.** A specific subset of N1 neutrophils, characterized by the expression of HLA-DR and CD86, can be induced by GM-CSF and IFN-γ [109]. These neutrophils are capable of activating antitumor adaptive immunity by interacting with CD8^+^ T cells and CD4^+^ T cells through MHC-TCR binding [109]. Conversely, the presence of GM-CSF and IL-6 induces another subset of TANs that express CCL4 and CCL3, facilitating the recruitment of macrophages and thereby promoting tumor metastasis [112]. Additionally, TANs exhibiting elevated levels of CD54 and CD11b, along with reduced CD62L expression, have been shown to secrete CXCL10, IL23a, and Arg1. These molecules, in conjunction with IL-12 secreted by macrophages, collaborate to stimulate the secretion of IFN-γ by unconventional αβ T cells, thereby eliciting a type I immune response against tumor cells [116]. Another subset of TANs expressing CD274 (PD-L1), which is differentiated by lactate, can interact with T cells and subsequently hinder their cytotoxicity against tumors [112, 131]. Furthermore, B cells exhibiting high levels of CD45 and B220, as well as low level of CD138, have been reported to be recruited by TANs to plasma cells exhibiting low levels of B220 and CD138, as well as high level of CD45, by contrast [123]. These TANs secrete the cytokine BAFF (BLyS) and the proliferation-inducing ligand APRIL, which not only contribute to B cells recruitment but also the IgM production, along with its switching to IgG or IgA [124]. Noticeably, apart from molecules such as cytokine BAFF (BLyS), APRIL, and IL-21, TNF-α is also reported to increase the movement and support the migration of B cells along with CXCL12 or CXCL13 [123, 132]. Besides, researchers have pointed out the important function of NETs as well as the direct contact of BAFF and BAFF-R in the B cells recruiting process [124]

### The function of NETs in the TME

NETs, composed of decondensed chromatin DNA filaments coated with granule proteins, can be induced by tumors through the secretion of various factors derived from tumors and infections. NETs are released by neutrophils and serve as an important mechanism for trapping pathogens with microbicidal activity by binding to these DNA structures [132]. However, their predominantly pro-tumorigenic role in cancer has recently gained attention. In addition to their involvement in tumor angiogenesis, NETs can promote cancer proliferation by mediating laminin remodeling and inducing immunosuppression. Moreover, NETs contribute to cancer-associated thrombosis, tumor intravasation, and metastasis by facilitating epithelial-to-mesenchymal transition, capturing CTCs, and increasing vascular permeability [133].

Tumors can induce the formation of NETs through the secretion of various tumor- and infection-derived factors, such as the overexpression of G-CSF commonly observed in cancer. Cutting off this potential cascade reaction cycle to suppress tumor activities is an intriguing concept worth considering [134]. Recent studies suggest that a cell-intrinsic program, regulated by the receptor CXCR2 and circadian cycle regulators, probably give rise to the alteration of neutrophil proteome in circulation, leading to the progressive loss of granule content and a decrease in NET-forming capacity [135]. Taking into account the molecular receptor CXCR2 and circadian cycle regulators could offer a more comprehensive approach to developing targeted therapies.

In tumor-associated aged neutrophils (CXCR4^+^CD62L^low^), induced by tumor-derived Nicotinamide Phosphoribosyl transferase (NAMPT), are capable of forming two types of NETs: mitochondria-dependent vital NETs, where SIRT1 induces the opening of mitochondrial permeability transition pore channels to release mitochondrial DNA, and traditional Cit-Histone H3-dependent fatal-NETs [136, 137]. This research highlights the potential of targeted therapy at the SIRT1-Naged-NETs axis in breast cancer lung metastasis [137]. Besides, researches have suggest that the NAMPT/SIRT pathway is implicated in angiogenesis as well as pro-tumorigenic effects of TANs [105], which can impact the prognosis of different types of cancers. SIRT3 has the capability to modulate endothelial cells, stimulating an increase in reactive oxygen species (ROS) production and regulating the hypoxia-inducible factor (HIF), ultimately culminating in the promotion of angiogenesis [138]. Therapeutic strategies targeting these pathways have been explored, but further investigation is needed to determine if this targeted therapy can positively affect other types of cancer metastasis and if other TAN phenotypes exhibit similar characteristics, allowing for more precise localization [139, 140].

**Fig. 5:**
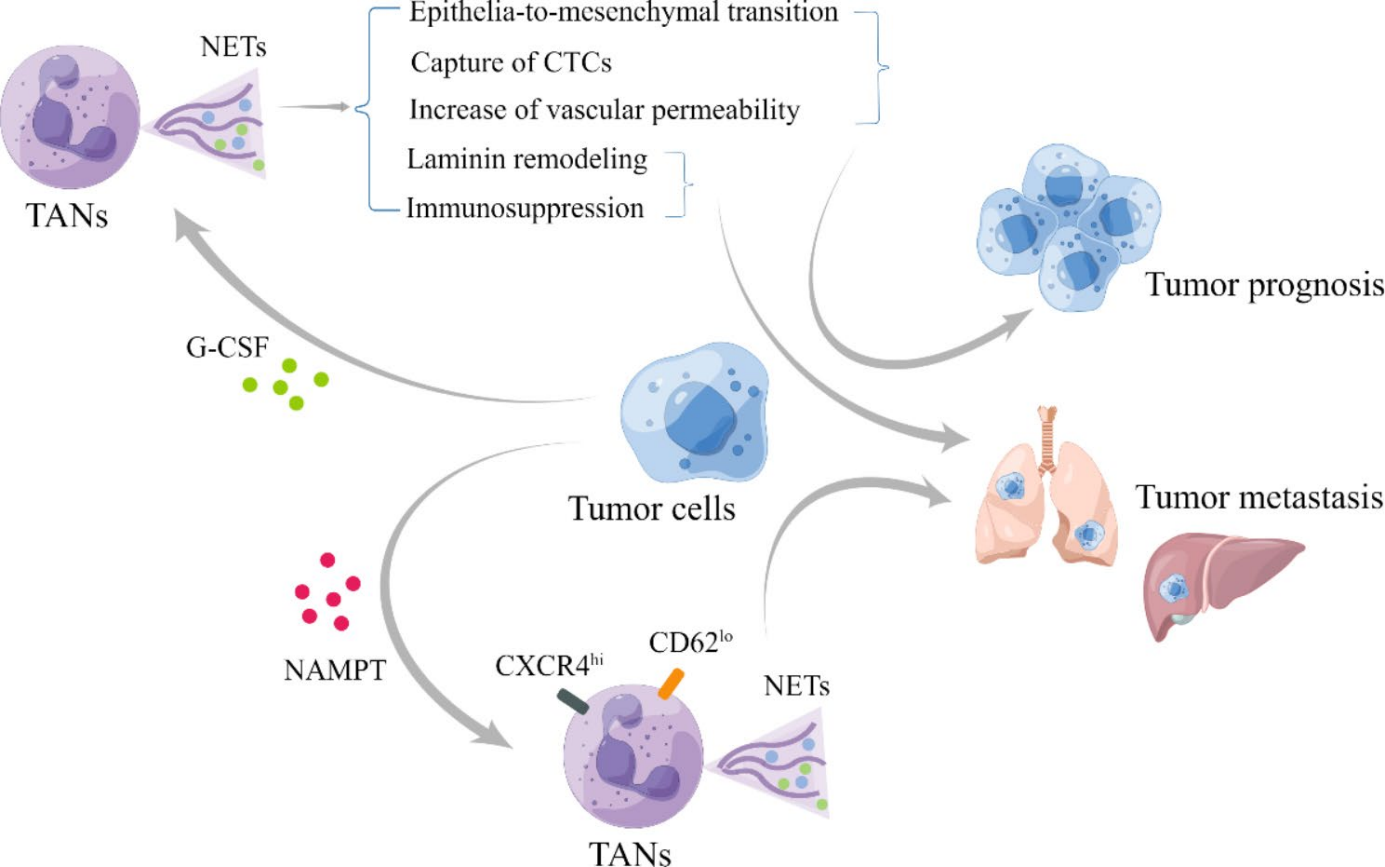
**The function of NETs in the TME.** Tumor cells possess the ability to secrete various molecules, including G-CSF and NAMPT. G-CSF can induce the formation of NETs, which exhibit diverse biomedical behaviors such as epithelial-to-mesenchymal transition, capture of CTCs, and increased vascular permeability that contribute to tumor prognosis [133]. However, they can also exhibit laminin remodeling and immunosuppressive behaviors that promote tumor metastasis [133]. On the other hand, NAMPT can stimulate the formation of aged neutrophil-derived NETs with high CXCR4 expression and low CD62L expression, consequently promoting tumor metastasis [137]

### Neutrophil clearance

After fulfilling their various and specific functions in circulation and the TME, neutrophils undergo a process of clearance and replenishment. This process involves a phenotypic drift of neutrophils, where their phenotypes change from the time they are released into the blood (fresh neutrophils) to the time they are cleared from circulation (aged neutrophils). The aging process emphasizes the importance of time as a crucial parameter for neutrophil clearance [141]. Neutrophil clearance can occur through various pathways, which are regulated to synchronize with pathogens, including tumor cells, in order to maintain a steady state in the body [141, 142]. Resident macrophages can eliminate neutrophils in peripheral tissues, while aged neutrophils in circulation can persistently be recruited back to the bone marrow in a CXCR4/CXCL12-dependent manner. During this process, the expression of CD62L decreases, while CXCR4 increases, as mentioned previously [36, 143, 144].

Although some phenotypes have been revealed, such as CD16^lo^ in immature neutrophils and CXCR4^hi^ and CD62L^lo^ in older neutrophils during human neutrophil senescence, which is thought to be the result of activation rather than a distinguishing marker in the growth program, it is important to conduct further explorations and establish a generally accepted classification of neutrophil subsets based on different ages [141].

Meanwhile, questions are being raised regarding the lifespan of TANs compared to other neutrophils and how this difference is regulated in the TME [33]. Studies have indicated that factors such as gastric cancer cell-derived exosomes (GC-Ex) can prolong the survival of neutrophils and induce the expression of inflammatory factors in these cells. However, further research is required to gain deeper understanding of these mechanisms [145]. The mechanisms underlying the recognition and clearance of distinct neutrophil subsets within the TME remain unknown. It is also unclear whether these neutrophil subsets undergo further differentiation after fulfilling their functions but before being eliminated. Researchers have discovered that this latter pathway involves the activation of pro-inflammatory signaling pathways, including Toll-like receptor (TLR)-dependent and MyD88-dependent recognition of signals [146]. Further investigation, particularly in pathological conditions such as the TME, may provide valuable insights for manipulating the presence and distribution of TANs in cancer treatment.

**Fig. 6:**
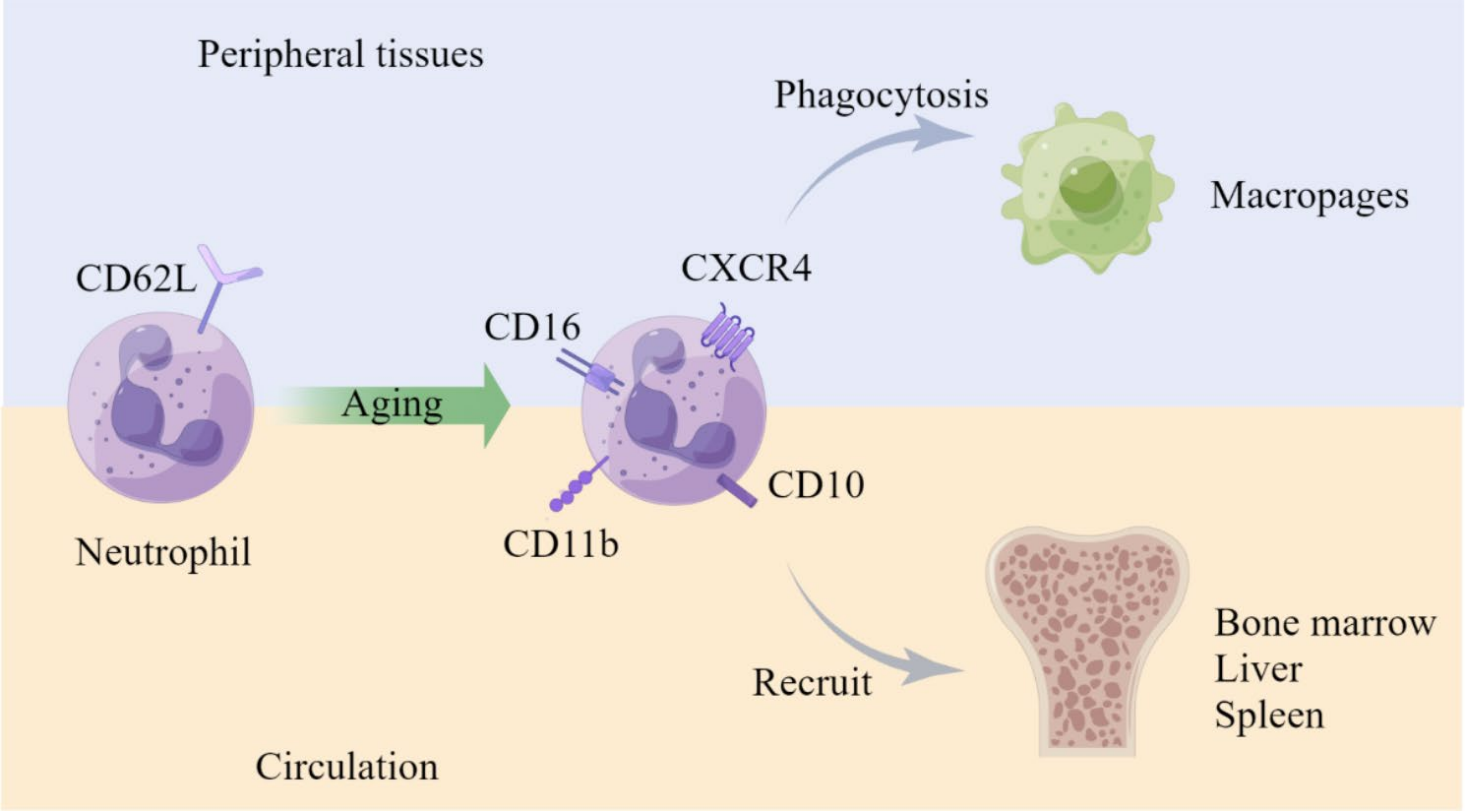
**Neutrophil clearance.** In cancer, the clearance of neutrophils is also mediated by key signaling molecules. This process exhibits distinct characteristics in both the tissue and circulation [36]. Aged neutrophils, which have a shorter lifespan, express increasingly high levels of CD16, CXCR4, CD10, and CD11b. They can either be recruited back into the bone marrow through a CXCR4/CXCL12-dependent pathway or be eliminated by macrophages in peripheral tissues [142–144]

### The future of neutrophils in cancer: Inspirations for diagnosis and treatment

#### The controversial correlation between neutrophils and prognosis

Numerous studies have elucidated the correlation between neutrophils and cancer prognosis, albeit with a predominant focus on peripheral blood neutrophils rather than TANs, primarily due to challenges in assessing the status (including number and subsets) of TAN infiltration [19]. Initial investigations highlighted a significant association between neutrophil-lymphocyte ratios (NLRs) and prognosis across several cancers, including prostate, colorectal, and pancreatic cancer [147–149]. In both early and advanced solid tumors, higher NLRs have consistently corresponded to poorer outcomes [147, 150]. Affirming this notion, Arnoud J Templeton and colleagues convincingly demonstrated a definitive link between elevated NLR and adverse OS in diverse solid tumors [151]. Nonetheless, the underlying mechanisms driving this phenomenon necessitate further exploration. What is the proportion of pathologic neutrophils among these circulating neutrophils in light of our previous discussion? which subset of these circulating neutrophils will become tumor infiltrating? How to identify them? These questions will guide future research endeavors.

Intriguing findings have emerged when investigating the infiltrating neutrophils within tumors. Higher TAN infiltration in the TME has been consistently associated with poorer outcomes in various cancers, such as renal cell carcinoma, head and neck cancers, and esophageal cancers [150]. However, contrasting results have been observed, as TANs can also be deemed a favorable prognostic factor, as evidenced in colorectal cancer [152]. Nevertheless, drawing directly predicting prognosis solely based on the numbers of infiltrating neutrophils may yield only partial conclusions, as the infiltration level varies across many aspects, such as location and stages. Some studies focused on the location of TAN infiltration have revealed that peritumoral infiltration is closely linked to improved prognosis [99] compared to intratumoral infiltration [153]. Additionally, the impact of cytokines involved in recruiting and regulating TANs, such as CSF, has been implicated, as the level of cytokines varies within different locations in the TME, potentially influencing the polarization of TANs [154]. Besides, Maria Rosaria Galdiero et al. reported a dramatic decrease in TAN density in Stage IV patients compared to Stage I-III in colorectal cancer, thereby impacting the effects of chemotherapy and finally resulting in a totally different prognosis [155]. Another study considered both stage and infiltrating location identified low TAN infiltration at the front of the tumor invasion site as an independent prognostic factor for poor prognosis in patients with early colon cancer [156]. Similar conclusions were drawn by Heng Zhang et al. regarding gastric cancer and the predicting value of TAN infiltrations in chemotherapy [157]. Furthermore, TANs have been shown to induce impaired antitumor immunity via the modulation of PD-L1/PD-1 signaling pathway, influencing the efficacy of PD-1 therapy in hepatocellular carcinoma (HCC) [154]. Therefore, it is evident from the aforementioned studies that the infiltration of neutrophils must be considered in conjunction with lots of variables beyond mere numbers, including tumor location and stage, when attempting to predict cancer prognosis. Additionally, the level of neutrophil infiltration may also impact the efficacy of chemotherapy or immunotherapy, thereby influencing overall prognosis.

As scientific research progresses, the underlying complexity of the controversial issue surrounding TANs has begun to unveil, highlighting their plasticity and heterogeneity. For instance, in pancreatic ductal adenocarcinoma (PDAC), higher TAN infiltration was initially believed to correlate with poorer OS [158, 159]. However, recent investigations have introduced a novel approach to assessing infiltration and have demonstrated the malleability of TANs, with the N1/N2 ratio emerging as a critical prognostic indicator [11, 13]. Collectively, these studies emphasize that relying solely on the quantification of TANs to evaluate prognosis is a limited strategy that can only yield partial outcomes. Future investigations on neutrophil prognosis will benefit from considering the infiltration level of specific subsets.

In addition, NETs have been identified as independent prognostic factors in cancer [160]. In breast cancer, the recruitment of neutrophils and the formation of NETs are mediated by the tumor-secreted protease cathepsin C, promoting lung metastasis and resulting in a poor prognosis [52]. Consequently, NETs are considered promising targets in tumor therapy. Currently, the markers H3Cit and MPO-DNA serve as valuable predictors for NET formation and are indicative of the prognosis in cancer patients [159]. Some studies have proposed that the detection of NETs and G-CSF in tumor biopsy tissues can assess the effect of targeted therapies in patients, although a definitive conclusion has not yet been reached [161–163].

An alternative perspective involves considering the correlation between neutrophils and prognosis in terms of metastasis. Within the circulation, neutrophils have been observed to transport metastatic cancer cells by forming cell clusters through interactions with endothelial cells [164]. These neutrophil-cancer cell clusters exhibit significant metastatic capacity [71], and play an important role in extracellular matrix remodeling and activation of stromal cells [165]. In lung cancer, tumor-derived exosomes can stimulate neutrophils to migrate to specific metastatic sites by activating Toll-like receptors 3 in alveolar cells [166], subsequently enabling them to acquire immunosuppressive functions [55, 167]. However, research exploring the connection between circulating tumor-promoting neutrophils and TANs remains limited. In the future, accurately identifying specific subsets of circulating tumor-promoting neutrophils and elucidating their origin and development will emerge as a prominent focus in this field. These aberrant circulating neutrophils have great potential to be therapeutic targets to inhibit tumor progression.

### The complex role of neutrophils in multiple treatment strategies

Neutrophils are closely linked to the clinical management of cancer and the benefits of several therapies, such as chemotherapy and radiotherapy. The numbers of circulating neutrophils and their infiltration status have been found to be associated with treatment outcomes.

In the context of chemotherapy, neutropenia is a common concern as it can impact treatment [168, 169]. Recombinant G-CSF or GM-CSF is often administered alongside chemotherapy to increase white blood cell counts, but there is limited research focusing on the possible modulation of neutrophils. It remains unclear whether the recruited neutrophils exert a pro- or anti-tumor effect, and whether there are differences between circulating neutrophils and those within the TME [170, 171]. The involvement of neutrophils in radiotherapy has also been investigated. In non-small cell lung cancer, the pre-treatment NLR is considered an important prognostic indicator of survival [172]. Studies have shown that radiotherapy can induce local sterile inflammation, leading to the recruitment and infiltration of neutrophils, which may subsequently transform into TANs [173]. Increased deposition of NETs has been observed in the TME of mice treated with radiation therapy for bladder cancer [174]. The use of G-CSF in radiation therapy has been shown to polarize TANs towards the N1 phenotype and enhance their anti-tumor activity [175]. However, Kellie et al. [176] have pointed out that the use of G-CSF in adjuvant therapy for breast cancer should be careful since it may promote metastasis. The effects of combination therapy involving radiotherapy/chemotherapy and cytokines such as G-CSF or other chemokines are still controversial and insufficiently documented.

The pivotal involvement of neutrophils in the realm of immunotherapy has been initially elucidated through several retrospective cohort studies. These studies have consistently demonstrated a significant association between elevated NLRs and a compromised prognosis following immune checkpoint inhibitors (ICIs) therapy in multiple cancers [177]. Notably, patients with melanoma subjected to treatment with the anti-CTLA-4 antibody, ipilimumab, exhibited a marked reduction in granulocyte levels, suggesting a potential dearth of synergistic interaction between CTLA4 and neutrophils [178]. On the other hand, He et al. [154] unveiled a heightened expression of PD-L1 in both intratumoral and peritumoral neutrophils, surpassing that observed in circulating neutrophil populations, among patients afflicted with HCC. These particular neutrophils suppressed the proliferation and activation of T cells, thereby underscoring their indispensability in the context of PD-1-based therapeutic interventions. However, intriguingly, the intratumoral neutrophil counts in melanoma patients subjected to treatment with the anti-PD-1 antibody, nivolumab, did not deviate significantly from those of non-users [179]. In the domain of PDAC, the utilization of nivolumab was associated with escalated infiltration density of TANs, which is correlated to worse OS outcomes [180]. Recent scientific endeavors have proffered a more nuanced perspective, positing that the hitherto established view may have been somewhat one-sided, and that certain specialized subsets of TANs exert a pronounced impact on the efficacy of immunotherapy. Jeremy et al. [181] compellingly demonstrated that effective immunotherapy engendered a transient escalation in the abundance of TANs. Notably, this investigation delineated a distinctive cohort of therapy-induced neutrophils that prominently exhibited an interferon gene signature, which is an attribute deemed indispensable for the immunotherapeutic regimens. In another study, researchers achieved remarkable eradication of melanoma via T-cell therapy in a murine model, despite the majority of melanoma cells lacking the target antigen Trp1, with the help of a subset of neutrophils that evinced the capability to secrete nitric oxide (NO), thereby effectively annihilating these immune-escaped tumor cells [179]. The precise ramifications of neutrophils in ICI-based therapeutic interventions is still controversial. However, we can assume that the efficacy of ICI treatment may be markedly influenced by specific TAN subsets. The exact mechanisms governing the interaction between ICIs and TANs in the TME are yet to be comprehensively investigated. Consequently, an accurate delineation and precise targeting of TAN subsets within the purview of ICI-based therapies undoubtedly hold promise as indispensable determinants of enhanced therapeutic efficacy.

### Treatment strategies targeting neutrophils

The burgeoning correlation between neutrophils and prognosis, along with their potential role in chemotherapy, radiotherapy, and immunotherapy, has sparked significant interest in the notion of directly targeting neutrophils as a promising treatment strategy. This emerging topic has garnered widespread attention [182, 183].

One potential therapeutic approach involves augmenting the number of circulating neutrophils and enhancing their biological function. Parenteral administration of recombinant human G-CSF has demonstrated efficacy in the treatment of severe neutropenia. G-CSF facilitates faster release of neutrophils from the bone marrow and amplifies their function [168]. However, it is worth noting that G-CSF treatment may also mobilize immature neutrophils from the bone marrow, and the role of these immature cells in the pathological context, whether in circulation or within the TME remains uncertain. Another way to increase the population of circulating neutrophils involves inhibiting the CXCL12-CXCR4 axis. For instance, the small molecule CXCR4 inhibitor Plerixafor exhibits potential for mobilizing hematopoietic stem cells, and the inhibition of CXCL12/CXCR4 signaling may serve as an adjuvant in the treatment of hematologic malignancies and solid tumors [183–185]. Additionally, there have been notable advancements in understanding TAN recruitment. Recent investigations indicate that IL-8 suppresses TAN accumulation in papillary thyroid carcinoma, although the precise phenotype of IL-8-activated TANs necessitates further exploration [182]. As the diversity of neutrophil subsets continues to be elucidated, the notion of simply mobilizing and increasing the number of neutrophils in the body is increasingly recognized as overly simplistic. Further research should focus on elucidating strategies to induce more anti-tumor phenotypes among the expanded neutrophil population.

Another feasible study to enhance the anti-tumor function of neutrophils is to directly enhance their functional responsiveness. Antagonizing various inhibitory receptors on neutrophils may facilitate the neutrophil-mediated anti-tumor function, thereby engendering a notion of neutrophil checkpoint blockade [186, 187]. Neutrophils share certain characteristics with other myeloid immune cells, enabling them to engage in intricate interactions and mutually reinforce each other’s anti-tumor functions. Several therapeutic approaches have sought to target the “don’t eat me” signal mediated by the interaction between SIRPα expressed on myeloid cells and CD47, which effectively prolongs the lifespan of neutrophils [188]. In vitro studies have demonstrated that neutrophils possess the capacity to eliminate antibody-opsonized cancer cells through trogocytosis [74], with neutrophil-mediated (ADCC ) serving as an efficacious mechanism for tumor eradication. Notably, owing to the selective expression of Fcα receptors on neutrophils, artificial IgA antibodies, instead of IgG antibodies, can elicit robust antibody-dependent cytotoxicity, thereby facilitating the eradication of tumor cells [189].

On the other hand, certain studies explore the inhibition of neutrophil function due to their pro-tumor phenotype, focusing on reducing neutrophil numbers or blocking their activation. Therapeutic strategies encompass the utilization of selectin antagonists, anti-integrin antibodies, CXCR1 inhibitors, CXCR2 inhibitors, leukotriene B receptor 1 (BLT1) inhibitors, C5a receptor inhibitors, and NETs inhibitors, aiming to neutralize neutrophil-derived molecules, block receptor activation, and impede signal transduction [190–194]. Notably, CXCR2 has emerged as a pivotal target in tumor treatment since it facilitates the recruitment and activation of certain myeloid cells [194]. Spleen tyrosine kinase (SYK) plays a key role in neutrophil β2 integrin and Fc receptor signaling pathways, and highly selective SYK inhibitors are currently being developed [195, 196]. In a mouse model of PDAC, lorlatinib has been shown to indirectly inhibit PDAC growth at primary and metastatic sites by modulating neutrophil development and inhibiting neutrophil-induced tumor growth within the TME [197]. Similarly, in patients with HCC, sorafenib promotes neutrophil infiltration into the tumor [198]. Consequently, the combination of tyrosine kinase inhibitors and depletion of TANs is believed to exert a more potent tumor-suppressive effect [197, 198]. Given the existing challenge of precisely targeting anti-tumor/pro-tumor neutrophil subsets and considering the prevailing dominance of N2-type TANs within the TME [11], it may be expedient to opt for strategies that reduce neutrophil numbers and inhibit their function within the current landscape of neutrophil-based therapeutic interventions.

The phenotype of TANs allows for their transition between anti-tumor and pro-tumor phenotypes, underscoring the need for therapeutic strategies aimed at restoring neutrophil function, rather than simply enhancing or inhibiting [199]. This polarization is thought to arise from the tumor-driven release of immature neutrophils from the bone marrow and the emergence of polymorphonuclear myeloid-derived suppressor cells (MDSCs) [200]. Consequently, current endeavors are focused on identifying key molecular players involved in MDSC formation. Notably, the inhibition of fatty acid transporter (FATP2) is a promising approach, as it can orchestrate the reprogramming of neutrophils and shift them towards an MDSC phenotype [201]. In a murine model of tumors, the inhibition of FATP2 with Lipofermata has demonstrated the ability to reduce tumor size by blocking the formation of MDSCs [202]. Furthermore, inhibitors targeting endoplasmic reticulum stress are believed to attenuate the MDSC function and formation, thereby contributing to the restoration of neutrophils to an anti-tumor phenotype [203]. From the perspective of MDSC modulation, the utilization of SYK inhibitors and other pharmaceutical agents to eliminate neutrophils and inhibit their activation has also been explored [204]. Undoubtedly, MDSCs, as representative markers of immunosuppressive subsets, are poised to emerge as a captivating research hotspot in the near future for targeting neutrophils.

**Table 1:** Clinical trials on targeting neutrophil relevant strategies in cancers

Purpose	Targets	Agents	Clinical Trials
Inhibition of recruitment and promotion of apoptosis	CXCR1/ CXCR2 inhibitor	Reparixin	NCT02370238
			NCT02001974
	TRAIL receptor agonists	Tigatuzumab	NCT01307891
		CS-1008	NCT01220999
		Mapatumumab	NCT01088347
		AMG 951	NCT00508625
		TRM-1	NCT00092924
	Anti- CD40 monoclonal antibody	CP-870, 893	NCT01103635
			NCT00607048
	CCR5 antagonists	Maraviroc	NCT03274804
			NCT01736813
	CD47–SIRPα inhibitors	Hu5F9-G4	NCT02216409
		IBI188	NCT03717103
		CC-90,002	NCT02367196
Inhibition of pro-tumor function	Neutrophil elastase inhibitors	Sivelestat	NCT01170845
	PI3K inhibitors	Buparlisib (a PI3Kδγ inhibitor)	NCT02194049
			NCT01629615
	PDE5 inhibitors	Sildenafil	NCT02544880
			NCT00752115
		Tadalafil	NCT01697800
	NSAIDs	Aspirin and ibuprofen (COX1 and COX2 inhibitors)	NCT01786200
		Celecoxib (a COX2 inhibitor)	NCT02429427
Enhancing the tumor-promoting function(Switch phenotype)	C/EBPα	MTL-CEBPA small activating RNA	NCT02716012
			NCT04105335
	TGF-β pathway inhibitors	Galunisertib	NCT01582269
			NCT01682187
			NCT03206177
			NCT02672475
			NCT02734160
			NCT02452008
		Fresolimumab (an anti- TGFβ monoclonal antibody)	NCT02581787
	STAT3 inhibitors	Napabucasin (BBI608)	NCT02753127
			NCT02358395
	β- Glucans	ImuCell WGP	NCT00682032
Increasing recruitment and longevity	G- CSF mimetics	Pegfilgrastim	NCT00035594
		YPEG- rhG-CSF (a long- acting form of pegfilgrastim)	NCT02005458
	Angiogenesis inhibitors	Plinabulin (an inhibitor of tubulin polymerization, as required for the formation of new blood vessels)	NCT00630110
			NCT02504489
			NCT03102606

### Discussion and perspectives

Neutrophils, with their relatively short lifespan, continuously differentiate from the bone marrow into various subsets, each possessing distinct functions In the context of tumor-related pathology, these subsets exhibit multifaceted functions, including angiogenesis, extracellular matrix remodeling, metastasis, and immunosuppression, exerting both direct and indirect anti-tumor effects, possibly via a complicated cellular network involving neutrophils, ultimately contributing to immune suppression within the TME [32, 33, 205, 206]. As commonly recognized, transcription and translation serve as fundamental links in comprehending neutrophil heterogeneity, wherein distinct neutrophil subsets undertake multiple functions while preserving the same genotype in an individual [30, 207, 208]. The classification and detection of these distinct phenotypes represent key research focal points in the field of cancer [209]. The introduction of the N1 and N2 neutrophil nomenclature in 2009 marked an initial endeavor to differentiate neutrophils into anti-tumor and pro-tumor subsets. However, this classification lacks the requisite specificity and precision to fully describe the heterogeneity of neutrophils [12]. Consequently, it is crucial to acknowledge the heterogeneity of TANs at different stages and comprehend their abnormal functions within the TME. With the heterogeneity of neutrophils widely recognized, further inquiries arise [209]. Our current understanding of subset classification remains incomplete, and it remains unclear whether these subsets arise from differentiation in the bone marrow, maturation in circulation, or reprogramming within the TME [38]. Precisely delineating the origin of TAN subsets and comprehending their complete lifespan could furnish researchers with a comprehensive understanding of TAN heterogeneity, enabling the optimization of timing for interventions in neutrophil-targeted immunotherapy and ultimately improving patient prognosis. Furthermore, a universally accepted definition and separation method for each neutrophil subset including TAN subsets, alongside deeper insights into their functions, is imperative [38]. With well-defined definitions, separation techniques, and purification methods for each neutrophil subset, particularly TAN subsets, patients may be one step closer to the promising realm of neutrophil immunotherapy, akin to the success witnessed with CAR-T cell therapy [85, 210–212].

## Conclusion

The heterogeneity of neutrophil subsets in the context of cancer exerts a pivotal influence on prognosis. Future endeavors must intimately delve into the complete lifespan of these neutrophils. The accurate identification of distinct neutrophil subsets and the precise targeting of key pro-tumor/anti-tumor subsets hold immense promise as therapeutic approaches.

## Data Availability

Not applicable.

## Abbreviations

Alox5: Arachidonic acid 5-lipid
ANGPT1: Angiopoietin1
ANGPT2: Angiopoietin2
APRIL: A Proliferation-Inducing Ligand
BAFF: B-cell activating factor
BLT1: Leukotriene B receptor 1
CSF: Colony stimulating factors
CTC: Circulating tumor cell
CXCL: C-X-C motif chemokine ligand
CXCR: Chemokine receptor
DAMP: Damage-associated molecular pattern
eNeP: early-stage neutrophil progenitor
FATP2: Fatty acid transporter 2
G-CSF: Granulocyte colony stimulating factor
GMP: Granulocyte-monocyte progenitor
HCC: Hepatocellular carcinoma
HIF: hypoxia-inducible factor
IFN-γ: Interferon-gamma
IL: Interleukin
LDNs: low-density neutrophils
MDSC: Myeloid-derived suppressor cell
MMP-9: Matrix metalloproteinase-9
NAMPT: nicotinamide phosphoribosyltransferase
NE: Neutrophil elastase
NETs: Neutrophil extracellular traps
NGAL: Neutrophil gelatinase-associated lipocalin
NLRs: Neutrophil-lymphocyte ratios
NPAs: neutrophil-platelet aggregates
PDAC: Pancreatic ductal adenocarcinoma
PGE2: Prostaglandin E2
preNeu: pre-neutrophil
RNS: Reactive nitrogen species
ROS: Reactive oxygen species
SYK: Spleen tyrosine kinase
TAN: Tumor-associated neutrophil
TAM: Tumor-associated macrophage
TGF-β: Transforming growth factor beta
TLR: Toll-like receptor
TME: Tumor microenvironment
TNF-α: Tumor necrosis factor-alpha
